# The predictive value of the ages and stages questionnaire in late infancy for low average cognitive ability at age 5

**DOI:** 10.1111/apa.16309

**Published:** 2022-03-03

**Authors:** Andrea K. Bowe, Jonathan Hourihane, Anthony Staines, Deirdre M. Murray

**Affiliations:** ^1^ INFANT Research Centre, Paediatric Academic Unit Cork University Hospital Cork Ireland; ^2^ Department of Paediatrics, School of Medicine University College Cork Cork Ireland; ^3^ Paediatrics Royal College of Surgeons in Ireland Dublin Ireland; ^4^ School of Nursing, Psychotherapy and Community Health Dublin City University Dublin Ireland

**Keywords:** ages and stages questionnaire, cognitive ability, early child development, early intervention, Kaufman brief intelligence test

## Abstract

**Aim:**

This retrospective, longitudinal study examined the predictive value of the ages and stages questionnaire (ASQ) in late infancy for identifying children who progressed to have low cognitive ability at 5 years of age.

**Methods:**

The ASQ was performed on 755 participants from the Irish BASELINE birth cohort at 24 or 27 months of age. Intelligence quotient was measured at age 5 with the Kaufmann Brief Intelligence Test, Second Edition, and low cognitive ability was defined as a score more than 1 standard deviation below the mean. The ASQ’s predictive value was examined, together with other factors associated with low cognitive ability at 5 years.

**Results:**

When the ASQ was performed at 24 or 27 months, the overall sensitivity for identifying low cognitive ability at 5 years was 20.8% and the specificity was 91.1%. Using a total score cut‐off point increased the sensitivity to 46.6% and 71.4% at 24 and 27 months, but specificity fell to 74.1% and 67.2%, respectively. After adjusting for ASQ performance, maternal education and family income were strongly associated with cognitive outcomes at 5 years.

**Conclusion:**

The ASQ did not detect the majority of children with low cognitive ability at age 5. Alternative methods need investigation.

**Abbreviations**
ASQ: ages and stages questionnaire
IQ: intelligence quotient
KBIT‐2: Kaufman brief intelligence test second edition
SD: standard deviation

**Key Notes**

- This study assessed whether using the Ages and Stages Questionnaire at 24 or 27 months of age identified children with low average cognitive ability at 5 years.
- The overall sensitivity and specificity of the ASQ for identifying children with low average cognitive ability at 5 years were 20.8% and 91.1%, respectively.
- Current developmental screening will not detect the vast majority of children who commence education with low average cognitive ability.

## 1 INTRODUCTION

Children with low average cognitive ability represent a substantial proportion of children with often unrecognised difficulties.^1^, ^2^ Early deficits in cognitive ability are associated with later adverse outcomes in many domains of life including mental and physical health, educational attainment, socio‐economic status and substance use.^3^, ^4^, ^5^, ^6^, ^7^ Despite the well‐established adverse impacts, approximately 13% of children whose cognitive ability falls into this lower range receive little attention in scientific literature and often little recognition in ordinary life.^2^

Defining this level of cognitive ability can be difficult. The International Classification of Diseases, 11th Edition clearly defines a disorder of intellectual development as one that is characterised by significantly below average intellectual functioning and adaptive behaviour that are approximately two or more standard deviations (SD) below the mean on appropriately normed, individually administered standardised tests.^8^ The intellectual functioning of some children is substantially below the population average, but they do not meet these criteria. They remain in a grey area between average intellectual functioning and disordered intellectual development. Children with more significant intellectual developmental disorders display marked deficits that are likely to be detected during routine developmental screening. However, children in the grey area may not display overt signs during infancy. Their cognitive difficulties may go unrecognised until they experience repeated failures or behavioural problems at school.^1^, ^7^, ^9^

Early interventions are best focused on the first five years of life, a fundamental period in cognitive development, and the aim of routine developmental screening is to identify infants who would benefit from such interventions.^10^ The American Academy of Pediatrics recommends that parent‐reported measures should be considered during routine developmental screening and the Ages and Stages Questionnaire (ASQ) is one example.^11^ The ASQ is a parent‐reported questionnaire designed to discriminate between children with developmental delays and typical development.^12^ The National Steering Group for the Revised Child Health Programme has recommended its use during the Irish 24‐month public health nursing assessment.^13^

Studies have suggested that the ability of the ASQ to predict below average childhood intelligence quotient (IQ) depends on the cohort and the cut‐offs used.^14^, ^15^ The ASQ has high specificity at a general population level and may effectively identify children not at risk of neurodevelopmental disorders.^16^ A significant shortcoming of many developmental screening tests is that they do not adequately account for the social environment to which the child will be exposed.^17^ Charkulak et al found that the performance of the ASQ for identifying children with low IQ is dependent on the maternal education level.^18^

This study examined the predictive value of the ASQ performed in late infancy for identifying those who progress to have low average cognitive ability at 5 years of age. We also assessed whether the 24‐month or 27‐month ASQs would have a better predictive value if an optimal cut‐off, based on the total score, was used. Finally, we examined other important factors associated with lower than average cognitive ability in early childhood.

## 2 METHODS

### 2.1 Participants

The study population consisted of mothers and infants from the Irish Cork BASELINE Birth Cohort Study, which started in 2008.^19^ BASELINE recruited 1583 of the 2183 participants (73%) from the Screening for Pregnancy Endpoints study, a multi‐centre prospective study of low‐risk, primiparous women. The other 600 were recruited from the postnatal wards of Cork University Maternity Hospital. The subjects were followed up at 2, 6, 12 and 24 months and 5 years. 1101 children completed the ASQ Third Edition (ASQ‐3) at either 24 or 27 months and 755 of these completed the Kaufmann Brief Intelligence Test, Second Edition (KBIT‐2) at the age of 5. These 755 participants formed the study cohort (Figure S1).

### 2.2 Measures

The ASQ is a parent‐completed questionnaire with 30 items covering 5 development domains: communication, gross motor, fine motor, problem solving and personal social skills.^12^ The score is based on the parental response, which may be “yes” (10 points), “not yet” (0 points) or “sometimes” (5 points), to a statement about the child’s activity. Parents are asked to try the activities with their child, if necessary, before answering. Total scores are calculated for each domain and missing answers may be replaced by the mean of the other answers, if there are no more than 2 missing items per domain. Age‐specific statistically derived cut‐off points have been established for onward referral. The ASQ has not been validated in Ireland, and onward referral for further professional assessment is made based on an age‐specific score, in any domain, that falls at least 2 SD below the mean in the reference population.^12^ The BASELINE study mothers received an age‐appropriate ASQ‐3 and were asked to complete it before their next assessment at the research facility: 468 received the 24‐month ASQ and 287 received the 27‐month ASQ. The cut‐offs for onward referral are outlined in Table S1.

The KBIT‐2 was administered by trained research nurses to measure cognitive ability at 5 years of age. It comprises three subtests: verbal knowledge and riddles, which measure verbal intelligence, and matrices, which measures non‐verbal intelligence. The verbal and non‐verbal scores are combined to provide a composite IQ. The Kaufmann test has been shown to be a reliable and valid measure of IQ in children and adults aged 4–90 years.^20^ Children who scored more than 1 SD below the cohort mean were categorised as “Low Cognitive Ability” and those who scored above this as “Average or Above Cognitive Ability”.

### 2.3 Child, maternal and sociodemographic variables

Child characteristics included gender and weeks of gestation. Maternal characteristics included age in years, smoking status in first trimester (smoker, non‐smoker), weekly units of alcohol during the first trimester and their body mass index in kg/m^2^. Socioeconomic characteristics included total years of schooling and income category (≥€106000, €85 000–€105 000, €64 000–€84 000, €43000–€63 000, €21 000–€42 000, <€21 000).

### 2.4 Statistical analysis

The statistical analysis was conducted using R version 4.0.3 (R Foundation, Vienna, Austria). The study population was described using counts and percentages for categorical data and means and standard deviations for continuous data. *T*‐tests were used to compare the mean ASQ scores between the cognitive ability groups. The predictive value of the ASQ was examined by calculating its sensitivity, specificity, positive and negative predictive values and their 95% confidence intervals (CI). Previous studies have reported sensitivities ranging from 28% to 77% for predicting low cognitive performance, with results varying by the cohort tested and cut‐offs used.^14^, ^15^

The pROC package in R was used to generate receiver operating characteristic curves and calculate optimal cut‐off points for the total ASQ score.^21^ Univariable and multivariable logistic regression was used to examine child, maternal and socioeconomic predictors of low cognitive ability at 5 years. The findings are reported in line with the Strengthening the Reporting of Observational Studies in Epidemiology guidelines.^22^

## 3 RESULTS

### 3.1 Description of sample

The study sample consisted of 755 children who underwent the ASQ at either 24 or 27 months and the KBIT‐2 at 5 years. Their characteristics are compared to those who were not included due to missing data or attrition (Table 1). Children in the study sample were more likely to have a higher birthweight, have older mothers with more schooling, coming from higher income backgrounds and have higher ASQ scores. The ASQs were completed at a mean age of 25.1 ± 1.1 months, but the results are presented separately for 24 and 27 months, as the cut‐off points and score distributions differ between the 2 questionnaires. The Kaufmann test was completed at a mean age of 60.8 ± 1.7 months. The mean composite IQ score was 104.3 and 13.4% scored more than 1 SD below this point.

**TABLE 1:** Characteristics of children in study population compared to those in BASELINE cohort not included in study

	Children included in study *n* = 755 (34.6%)	Children not included *n* = 1428 (65.4%)	*p*‐value
	*n* (%)	*n* (%)	
*Child characteristics*			
Male	381 (50.5)	719 (50.4)	
Female	374 (49.5)	709 (49.6)	.996
Birthweight (g)	3538.3	3454.4	<.001
Gestational age (weeks)	39.60	39.40	.003
*Maternal characteristics*			
Maternal age (years)	30.8	29.5	<.001
*Smoking status 1st trimester*			
Smoker	126 (21.4)	213 (23.0)	
Non‐smoker	462 (78.6)	712 (77.0)	.003
BMI	25.0	24.8	.577
*Socioeconomic characteristics*			
Total years schooling	13.4	13.2	<.001
University completed			
Yes	428 (56.7)	631 (52.8)	
No	327 (43.3)	565 (47.2)	.099
*Accommodation type*			
Own house/flat	601 (79.6)	795 (66.5)	
Private rental	124 (16.4)	329 (27.5)	
Council/government rental	12 (1.6)	36 (3.0)	
Other	18 (2.4)	36 (3.0)	<.001
*Income category*			
<21k	27 (3.7)	129 (11.3)	
21–42k	126 (17.2)	235 (20.6)	
43–63k	169 (23.1)	267 (23.4)	
64–84k	189 (25.8)	226 (19.8)	
85–105k	112 (15.3)	162 (14.2)	
≥106k	110 (15.0)	123 (10.8)	<.001
*Ages and stages questionnaire*			
Completed 24 months	468 (62.0)	254 (62.3)	
Completed 27 months	287 (38.0)	154 (37.7)	1.000
Total ASQ score – mean (SD)	254.9 (31.0)	246.3 (44.9)	<.001
Total ASQ score – median (IQR)	260 (35)	255 (45)	.044
*Fail in ASQ domain*			
Communication	17 (2.5)	27 (6.7)	<.001
Fine motor	18 (2.4)	24 (6.2)	.002
Gross motor	20 (2.6)	15 (3.7)	.412
Problem solving	24 (3.2)	23 (5.9)	.040
Social skills	25 (3.3)	33 (8.3)	<.001
At least one domain	79 (10.5)	70 (18.6)	<.001
*KBIT‐2 IQ test*			
Composite IQ score – *n*, mean (SD)	755, 104.6 (8.7)	315, 103.6 (9.0)	.098

Note

*p*‐values calculated based on chi‐square tests for categorical variables and Welch two sample *t*‐tests for continuous variables.

We examined the mean ASQ scores for those with and without low cognitive ability at 5 years of age (Table S2) and the predictive value of the ASQ (Table 2). Children with low cognitive ability had significantly lower overall ASQ scores, with a mean difference of 16.6 points (95% CI 7.4–25.7) for the 24‐month ASQ and 24.8 points (95% CI 8.1–41.5) for the 27‐month ASQ. The ASQ had a sensitivity of 20.8% (95% CI 13.6%–30.2%) for identifying children with low cognitive ability at 5 years and a high specificity of 91.1% (95% CI 88.6%–93.2%) for identifying those not at risk.

**TABLE 2:** Predictive value of ASQ at 24/27 months for identifying children with low cognitive ability at age 5 years

	Sensitivity	Specificity	PPV	NPV
	% (95% Confidence Interval)			
One fail criterion	20.8 (13.6–30.2)	91.1 (88.6–93.2)	26.6 (17.6–37.9)	88.2 (85.5–90.5)
Fail communication	6.9 (3.1–14.2)	98.0 (96.5–98.9)	35.0 (16.3–59.1)	87.2 (84.5–89.5)
Fail gross motor	7.9 (3.7–15.5)	98.2 (96.7–99.0)	40.0 (20.0–63.6)	87.4 (84.5–89.6)
Fail fine motor	0.9 (0.5–6.2)	97.4 (95.8–98.4)	5.6 (0.2–29.4)	86.4 (83.7–88.8)
Fail problem solving	7.9 (3.7–15.5)	97.4 (95.8–98.4)	32.0 (15.7–53.6)	87.3 (84.6–89.6)
Fail social skills	10.9 (5.8–19.0)	97.9 (96.4–98.8)	44.0 (25.0–64.7)	87.7 (85.0–89.9)

Abbreviations: NPV, negative predictive value; PPV, positive predictive value.

**FIGURE 1:**
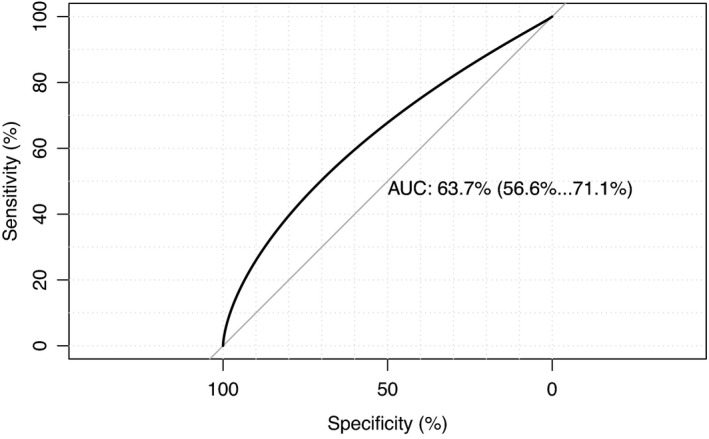
Receiver operating characteristic curve for 24‐month ASQ to predict low average cognitive ability at age 5 years. ROC curve performed using total ASQ score at 24 months for the prediction of composite IQ score <1standard deviation below cohort mean at age 5 years. A smoothed ROC curve was generated using the “pROC” package in R. Analysis includes *n* = 394 control and *n* = 73 cases. The area under the curve was 0.637, 95% confidence interval 0.637–0.709

The receiver operating characteristic curves for the 24 and 27 months ASQ are shown with their respective area under the curve values (Figures 1 and S2). At 24 months, a total ASQ cut‐off of 249.5 increased the sensitivity to 46.6% (95% CI 35.6%–57.5%), but reduced the specificity to 74.1% (95% CI 69.8%–78.2%). At 27 months, a total ASQ cut‐off of 244.5 increased the sensitivity to 74.1% (95% CI 53.6%–89.3%) and reduced the specificity to 67.2% (95% CI 61.4%–72.9%).

The results of the univariable and multivariable logistic regression analyses are shown in Table 3. After the data were adjusted for maternal and socioeconomic factors, those who failed at least one ASQ domain were 2.9 times more likely (adjusted odds ratio (AOR) 2.94, 95% CI 1.51–5.55, *p* < .001) to have low cognitive ability at 5 years. Other significant predictors were gestational age, weekly units of alcohol in the first trimester, total years of schooling and income. After adjustment, a 1‐year increase in maternal schooling was associated with a 40% reduction in the risk of low cognitive ability at 5 years. Children in the lowest income category were 5.0 times more likely (AOR 5.01, 95% CI 1.18–21.47, *p* = .027) to experience low cognitive ability at 5 years than those in the highest category.

**TABLE 3:** Univariable and multivariable logistic regression model examining factors associated with low cognitive ability at age 5

	Low *n* = 101	Average or above *n* = 653	Odds ratio (95% CI)	*p*‐value	Adjusted odds ratio (95% CI)	*p*‐value
*Child characteristics*						
ASQ one fail	21 (20.8)	58 (8.9)	2.69 (1.53–4.61)	<.001	2.96 (1.51–5.56)	.001
Gender – female	44 (43.6)	330 (50.5)	0.76 (0.49–1.15)	.193	1.08 (0.64–1.82)	.781
Birthweight – grams	3,497.8	3,544.8	1.00 (1.00–1.00)	.368		
Gestational age (weeks)	39.4	39.6	0.87 (0.76–1.01)	.052	0.84 (0.71–0.99)	.036
*Maternal characteristics*						
Maternal age – years	30.5	30.8	0.98 (0.92–1.04)	.457	1.02 (0.95–1.09)	.593
Smoking status						
Non‐smoker	54 (71.1)	407 (79.6)	Ref		Ref	
Smoker	22 (28.9)	104 (20.4)	1.59 (0.91–2.71)	.091	1.11 (0.59–2.01)	.742
Weekly units of alcohol 1st trimester	6.1	4.1	1.06 (1.02–1.10)	.003	1.06 (1.02–1.11)	.004
BMI	25.8	24.8	1.05 (1.00–1.11)	.064	1.03 (0.97–1.09)	.267
*Socioeconomic characteristics*						
Total years schooling	13.2	13.4	0.68 (0.52–0.89)	.004	0.69 (0.49–0.96)	.026
*Income category*						
>106k	9 (9.2)	101 (15.9)	Ref		Ref	
85–105k	10 (10.2)	102 (16.1)	1.10 (0.43–2.88)	.842	1.72 (0.59–5.41)	.325
64–84k	25 (25.5)	164 (25.8)	1.71 (0.79–4.01)	.189	1.93 (0.74–5.65)	.198
43–63k	26 (26.5)	143 (22.5)	2.04 (0.95–4.78)	.080	2.29 (0.89–6.72)	.103
21–42k	21 (21.4)	105 (16.5)	2.24 (1.01–5.38)	.055	2.49 (0.86–7.88)	.100
<21k	7 (7.1)	20 (3.1)	3.93 (1.27–11.82)	.015	5.01 (1.18–21.47)	.027

Note

95% CI, 95% Confidence Interval; BMI, body mass index measures in kg/m^2^; *p*, *p*‐value.

## 4 DISCUSSION

Our main aim was to examine the predictive value of the ASQ in late infancy for identifying children in the general population who progress to have low cognitive ability at 5 years of age. When the onward referral criterion of failing in a single domain was used, the overall sensitivity was 20.8%. The specificity, as previously documented,^16^ was 91.1%, making it a useful tool for identifying those not at risk. While the ASQ may identify children with significant domain‐specific developmental delay, for the purpose of identifying children with low cognitive ability in childhood, the ASQ at 24 or 27 months has an unacceptably low sensitivity, missing 80% of children who may benefit from earlier intervention.

The second aim was to determine whether an optimal cut‐off point, based on the total ASQ score, could improve the tool’s predictive value. Total ASQ scores are not currently used for onward referral, but studies suggest this may be a useful additional measure.^15^, ^23^ Using optimal cut‐off points of 249.5 and 244.5 on the 24‐month and 27‐month ASQs improved sensitivity to 46.6% and 71.4%, respectively, but reduced specificity. In population‐based screening, a careful balance must be struck between the sensitivity and specificity of a test. This improved sensitivity would mean that almost half of all children who progress to low cognitive ability at age 5 could be identified at 24 months. However, the accompanying reduction in specificity would mean that at 24 months approximately 1 in every 4 children who screened positive on the ASQ would be a false positive. This has significant ethical and societal implications. Labelling a child incorrectly as at risk of low cognitive ability may result in stigmatisation or may lead to lower expectations of ability which can adversely affect evolving capabilities.^24^ Additionally, a criterion for implementing any population‐based screening programme is that there must be an available course of action with regard to further diagnosis, treatment or intervention.^25^ Implementing this cut‐off could threaten to overwhelm the finite resources dedicated to early intervention programmes for at risk children. Implications would be similar with implementation of the 27‐month total score cut‐off.

The third aim was to examine the child, maternal and socioeconomic characteristics associated with low cognitive ability at 5 years. The findings were consistent with studies that have demonstrated strong, consistent associations between families’ socioeconomic characteristics and children’s cognitive outcomes.^18^ The odds of low cognitive ability were significantly higher for children whose mothers had lower levels of education and who were from lower income backgrounds. This finding again highlights the pivotal role socioenvironmental factors have in the cognitive development of a child.

This study investigated one potential screening method, the ASQ, for the purpose of early identification of children who progress to have low cognitive ability. It was found that, as it is currently used, this tool will not identify the majority of children with low cognitive ability at school entry. Other tools exist for identifying children at risk of low cognitive ability, the most widely used being the Bayley Scales of Infant Development (Bayley). This developmental assessment tool is usually administered by psychologists, paediatricians or physiotherapists and takes 30–70 min to complete, with extra time needed for scoring.^26^ It is resource intensive and is generally used for high risk populations, such as preterm or low birth weight infants.^27^ Studies of its use in the general population are limited, but evidence suggests that if it was performed in early life, particularly the first 24 months, it would have poor to modest correlations with later cognitive ability.^28^

Our study has limitations. The research question focussed on the use of the ASQ at a single time point in late infancy, but the authors acknowledge that developmental assessment should be a continuous process performed at multiple time points throughout infancy and childhood. The outcome was cognitive ability at age 5, but it is well recognised that the social and emotional competence of a child at this age are equally important determinants of outcomes in childhood.^29^ Cohort studies are subject to selection bias, and those who participate tend to systematically differ from those who do not.

## 5 CONCLUSION

Studies have shown that the ASQ has acceptable performance characteristics for identifying significant neurodevelopmental disorders. However, for the purpose of identifying children in the general population who progress to have low average cognitive ability at 5 years of age, this study found it is not a useful tool. Alternative methods of early identification are required. Further research into a systematic and robust method of identifying high‐risk children, based on environmental, genetic, biological, social and demographic factors, as well as the interactions between these factors, should be a public health priority.

## CONFLICTS OF INTEREST

The authors have no conflicts of interest to declare.

## INSTITUTIONAL REVIEW BOARD STATEMENT

Research objectives and measurements in the BASELINE birth cohort were conducted according to the guidelines laid down in the Declaration of Helsinki, and all procedures were approved by the Clinical Research Ethics Committee of the Cork Teaching Hospitals [ref ECM5(9) 01/07/2008]. The Cork BASELINE birth cohort study is registered at the United States National Institutes of Health Clinical Trials Registry (http://www.clinicaltrials.gov), ID: NCT01498965.

## INFORMED CONSENT

Informed consent was obtained from all participants in the BASELINE birth cohort study.

## Supporting information

Supplementary Material

## Data Availability

The BASELINE data are not currently publicly available due to ethical restrictions. Application for the use of the data in collaborative and ethically approved projects can be made through the INFANT centre website or by contacting infant@ucc.ie.

## References

[1] Wieland J , Zitman FG . It is time to bring borderline intellectual functioning back into the main fold of classification systems. Bjpsych Bull. 2016 Aug;40(4):204‐206. 27512590 10.1192/pb.bp.115.051490 PMC4967780

[2] Nouwens PJG , Lucas R , Embregts PJCM , van Nieuwenhuizen C . In plain sight but still invisible: a structured case analysis of people with mild intellectual disability or borderline intellectual functioning. J Intellect Dev Disabil [Internet]. 2017 Jan 2 [cited 2021 Feb 20];42(1):36‐44. Available from https://www.tandfonline.com/doi/full/ 10.3109/13668250.2016.1178220

[3] Deary IJ , Strand S , Smith P , Fernandes C . Intelligence and educational achievement. Intelligence. 2007 Jan 1;35(1):13‐21.

[4] Wraw C , Deary IJ , Gale CR , Der G . Intelligence in youth and health at age 50. Intelligence [Internet]. 2015 Nov 1 [cited 2020 Aug 6];53:2‐32. doi:10.1016/j.intell.2016.06.005 PMC4659286 26766880

[5] Schijven EP , Didden R , Otten R , Poelen EAP . Substance use among individuals with mild intellectual disability or borderline intellectual functioning in residential care: examining the relationship between drinking motives and substance use. J Appl Res Intellect Disabil. 2019 Jul;32(4):871‐878. 30844128 10.1111/jar.12578 PMC6850364

[6] Alesi M , Rappo G , Pepi A . Emotional profile and intellectual functioning. SAGE Open [internet]. 2015 Jul 10 [cited 2020 Nov 10];5(3). doi:10.1177/2158244015589995

[7] Fernell E , Ek U . Borderline intellectual functioning in children and adolescents—insufficiently recognized difficulties. Acta Paediatr. 2010 May;99(5):748‐753. 20146756 10.1111/j.1651-2227.2010.01707.x

[8] World Health Organisation . ICD‐11—Mortality and Morbidity Statistics [Internet]. [cited 2021 Apr 18]. Available from https://icd.who.int/browse11/l‐m/en#/http%3A%2F%2Fid.who.int%2Ficd%2Fentity%2F605267007

[9] Karande S , Kanchan S , Kulkarni M . Clinical and psychoeducational profile of children with borderline intellectual functioning. Indian J Pediatr. 2008 Aug;75(8):795‐800. 18581071 10.1007/s12098-008-0101-y

[10] Tierney AL , Nelson CA . Brain development and the role of experience in the early years. Zero Three [Internet]. 2009 Nov 1 [cited 2020 Dec 10];30(2):9‐13. Available from: http://www.ncbi.nlm.nih.gov/pubmed/23894221 23894221 PMC3722610

[11] Council on Children With Disabilities; Section on Developmental Behavioral Pediatrics; Bright Futures Steering Committee; Medical Home Initiatives for Children With Special Needs Project Advisory Committee . Identifying infants and young children with developmental disorders in the medical home: an algorithm for developmental surveillance and screening. Pediatrics. 2006 Jul;118(1):405‐420. doi:10.1542/peds.2006-1231 16818591

[12] ASQ‐3 Technical Report [Internet]. [cited 2020 Aug 14]. Brookes Publishing; 2009. Available from: https://agesandstages.com/wp‐content/uploads/2019/08/ASQ‐3‐Technical‐Appendix_web.pdf

[13] Health Service Executive . The Nurture Programme. National Guideline on the Use of the Ages and Stages Questionnaire for Developmental Screening of Children Between 1 and 66 Months of Age [cited 2021 Oct 07]. Available from: https://www.hse.ie/eng/health/child/nurture/asq3national‐guideline.pdf

[14] Schonhaut BL , Pérez RM , Castilla FA , Castro MS , Salinas AP , Armijo RI Predictive value of ages & stages questionnaires for cognitive performance at early years of schooling. Rev Chil Pediatr. 2017;88(1):28‐34. 28288224 10.1016/j.rchipe.2016.08.008

[15] Charkaluk M‐L , Rousseau J , Calderon J , et al. Ages and stages questionnaire at 3 years for predicting IQ at 5–6 years. Pediatrics. 2017 Apr;139(4):5‐6. 10.1542/peds.2016-2798 28360034

[16] Lamsal R , Dutton DJ , Zwicker JD . Using the ages and stages questionnaire in the general population as a measure for identifying children not at risk of a neurodevelopmental disorder. BMC Pediatr [internet]. 2018 Apr 3 [cited 2020 Aug 21];18(1):122. Available from https://bmcpediatr.biomedcentral.com/articles/ 10.1186/s12887-018-1105-z PMC5883588 29614989

[17] Eriksen H‐LF , Kesmodel US , Underbjerg M , Kilburn TR , Bertrand J , Mortensen EL . Predictors of intelligence at the age of 5: family, pregnancy and birth characteristics, postnatal influences, and postnatal growth. PLoS One [Internet]. 2013 Nov 13;8(11):e79200. doi:10.1371/journal.pone.0079200 24236109 PMC3827334

[18] Charkaluk ML , Rousseau J , Calderon J , et al. EDEN mother‐child cohort study group. Ages and stages questionnaire at 3 years for predicting IQ at 5–6 years. Pediatrics. 2017 Apr;139(4):e20162798. doi:10.1542/peds.2016-2798 28360034

[19] O’Donovan SM , Murray DM , Hourihane JOB , Kenny LC , Irvine AD , Kiely M . Cohort profile: the cork BASELINE Birth cohort study: babies after SCOPE: evaluating the longitudinal impact on neurological and nutritional endpoints. Int J Epidemiol [Internet]. 2015 Jun 1 [cited 2021 Feb 12];44(3):764‐775. doi:10.1093/ije/dyu157 25102856

[20] Bain SK , Jaspers KE . Test review: review of Kaufman brief intelligence test, second edition. J Psychoeduc Assess [Internet]. 2010 Apr 21 [cited 2021 Feb 12];28(2):167‐174. Available from http://journals.sagepub.com/doi/ 10.1177/0734282909348217

[21] Robin X , Turck N , Hainard A , et al. pROC: an open‐source package for R and S+ to analyze and compare ROC curves. BMC Bioinformatics. 2011;12:77. http://www.biomedcentral.com/1471‐2105/12/77/ doi:10.1186/1471-2105-12-77 21414208 PMC3068975

[22] Elm EV , Altman DG , Egger M , Pocock SJ , Gøtzsche PC , Vandenbroucke JP . Strengthening the reporting of observational studies in epidemiology (STROBE) statement: guidelines for reporting observational studies. BMJ. 2007;335:806. doi:10.1136/bmj.39335.541782.AD 17947786 PMC2034723

[23] Halbwachs M , Muller J‐B , Nguyen The Tich S , et al. Usefulness of parent‐completed ASQ for neurodevelopmental screening of preterm children at five years of age. PLoS One. 2013;8(8):e71925. pmid:24014166. 24014166 10.1371/journal.pone.0071925 PMC3754941

[24] McKown C , Weinstein RS . Teacher expectations, classroom context, and the achievement gap. J Sch Psychol. 2008 Jun;46(3):235‐261. doi:10.1016/j.jsp.2007.05.001. Epub 2007 Jun 20 PMID: 19083359. 19083359

[25] Dobrow MJ , Hagens V , Chafe R , Sullivan T , Rabeneck L . Consolidated principles for screening based on a systematic review and consensus process. CMAJ. 2018 Apr 9;190(14):E422‐E429. doi:10.1503/cmaj.171154. PMID: 29632037; PMCID: PMC5893317 29632037 PMC5893317

[26] Balasundaram P , Avulakunta ID . Bayley scales of infant and toddler development. [Updated 2021 Nov 24]. In: StatPearls [Internet]. StatPearls Publishing; 2022 Jan‐. Available from https://www.ncbi.nlm.nih.gov/books/NBK567715/ 33620792

[27] Liu T‐Y , Chang J‐H , Peng C‐C , et al. Predictive validity of the Bayley‐III cognitive scores at 6 months for cognitive outcomes at 24 months in very‐low‐birth‐weight infants. Front Pediatr. 2021 May;7(9):e638449. doi:10.3389/fped.2021.638449. PMID: 34026684; PMCID: PMC8138438 PMC8138438 34026684

[28] Månsson J , Stjernqvist K , Serenius F , Ådén U , Källén K . Agreement between Bayley‐III measurements and WISC‐IV measurements in typically developing children. J Psychoeducational Assess. 2019;37(5):603‐616. doi:10.1177/0734282918781431

[29] Miller MM , Goldsmith HH . Profiles of social‐emotional readiness for 4‐year‐old kindergarten. Front Psychol. 2017 Jan;31(8):132. doi:10.3389/fpsyg.2017.00132. PMID: 28197124; PMCID: PMC5281560 PMC5281560 28197124

