# Supplementary material for: The predictive value of the ages and stages questionnaire in late infancy for low average cognitive ability at age 5
Source: Acta Paediatr. 2022 Mar 3;111(6):1194–200. doi: 10.1111/apa.16309 (PMC9314849; doi:10.1111/apa.16309)

**Supplementary Material**

**Supplemental Figure S1. Flowchart of BASELINE Study Population**

Recruited to BASELINE birth cohort study n= 2,183

Consent withdrawn n=140 Lost to follow up n=82

Completed 2-month questionnaire n=1,961

Consent withdrawn n=41 Lost to follow up n=84

Completed 6-month questionnaire n=1,836

Consent withdrawn n=23 Lost to follow up n=69

Completed 12-month questionnaire n=1,744

Consent withdrawn n= 19 Lost to follow up n=108

Completed 24-month questionnaire n=1,617

Missing ASQ data n= 416 Completed ASQ at 22 months n=1 Completed ASQ at 33 months n=1

Completed 24/27 month ASQ n= 1,101

Missing IQ data at Age 5 n= 346

Kaufman IQ Test Completed at Age 5 and complete ASQ data n=755

Supplemental Table S1. ASQ Questionnaire Cut Offs Applied

|  | Communication | Gross Motor | Fine Motor | Problem Solving | Social skills |
| --- | --- | --- | --- | --- | --- |
| 24 months | <25.17 | <38.07 | <35.16 | <29.78 | <31.54 |
| 27 months | <24.02 | <28.01 | <18.42 | <27.62 | <25.31 |

Supplemental Table S2. Mean 24-month and 27-month ASQ Scores By Cognitive Ability

|  | Low Cognitive Ability | Average/above Cognitive Ability | Mean Difference | p-value |  | Low Cognitive Ability | Average/above Cognitive Ability | Mean Difference | p-value |
| --- | --- | --- | --- | --- | --- | --- | --- | --- | --- |
|  | n=73 | n=395 |  |  |  | n=28 | n=260 |  |  |
| 24-month ASQ | Mean (SD) | Mean (SD) | Mean Difference (95% CI)^^^ | p | 27-month ASQ | Mean (SD) | Mean (SD) | Mean Difference (95% CI)^^^ | p |
| Total ASQ | 244.6 (37.5) | 261.2 (27.2) | 16.6 (7.4-25.7) | 0.001 | Total ASQ | 226.4 (42.2) | 251.3 (29.9) | 24.8 (8.1-41.5) | 0.005 |
| Communication | 50.5 (12.6) | 55.6 (9.0) | 5.1 (2.0-8.2) | 0.001 | Communication | 50.7 (10.9) | 56.8 (6.1) | 6.1 (1.8-10.4) | 0.007 |
| Gross Motor | 53.4 (10.6) | 55.7 (6.2) | 2.3 (-0.2-4.9) | 0.072 | Gross Motor | 48.9 (13.1) | 50.5 (9.7) | 1.6 (-3.6-6.8) | 0.527 |
| Fine Motor | 49.4 (6.4) | 51.8 (7.3) | 2.4 (0.8-4.1) | 0.005 | Fine Motor | 36.9 (11.3) | 44.3 (12.3) | 7.6 (2.8-11.9) | 0.003 |
| Problem Solving | 43.9 (11.7) | 47.4 (9.7) | 3.5 (0.6-6.4) | 0.017 | Problem Solving | 46.0 (11.8) | 52.2 (8.0) | 6.2 (1.5-10.9) | 0.011 |
| Social Skills | 47.5 (11.1) | 50.7 (8.5) | 3.2 (0.5-5.9) | 0.023 | Social Skills | 43.9 (10.5) | 47.5 (9.1) | 3.5 (-0.6-7.7) | 0.095 |

95% CI, 95% Confidence Interval; p, p-value

Supplemental Figure S2. Receive Operating Curve for 27-month ASQ to predict low cognitive ability at age 5 years


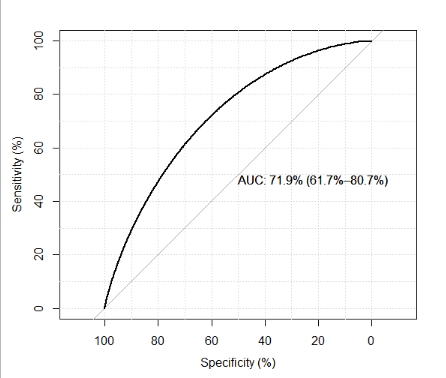

Supplement: Supplementary file 1 — Supplementary Material [file APA-111-1194-s001.docx]
